# Hybrid palliation of interrupted aortic arch in a high-risk neonate

**DOI:** 10.4103/0974-2069.64360

**Published:** 2010

**Authors:** Mohsen Karimi, Ahmed Farouk, Alex Golden, Robert Gilkeson

**Affiliations:** Division of Pediatric Cardiothoracic Surgery, Medical College of Georgia, Children's Medical Center, Augusta, Georgia, USA; 1 Pediatric Cardiothoracic Surgery, Rainbow Babies and Children's Hospital, Case Western Reserve School of Medicine, Cleveland, Ohio, USA; 2 Pediatric Cardiology, Rainbow Babies and Children's Hospital, Case Western Reserve School of Medicine, Cleveland, Ohio, USA; 3 Pediatric Radiology, Rainbow Babies and Children's Hospital, Case Western Reserve School of Medicine, Cleveland, Ohio, USA

**Keywords:** Acyanotic congenital heart disease, hybrid procedure, interrupted aortic arch

## Abstract

We report a case of a high-risk neonate with interrupted aortic arch (IAA) and ventricular septal defect who underwent a successful hybrid palliative procedure using a ductal stent and bilateral branch pulmonary artery banding. This case represents not only a successful use of hybrid approach in high-risk neonates with IAA, but also introduces an alternative and safe access for ductal stent insertion through the right ventricular infundibulum.

## INTRODUCTION

Interrupted aortic arch (IAA) is an uncommon congenital malformation characterized by lack of continuity between the ascending aorta and aortic arch. Since the introduction of prostaglandins (PGE1) in 1976, the preoperative management and operative results of neonates born with this complex cardiovascular disease have improved dramatically. However, interrupted aortic arch repair continues to be associated with substantial peri-operative morbidity and mortality primarily attributable to low birth weight and gestational age, presence of left ventricular outflow tract obstruction, hypoplastic aortic annulus or aortic arch hypoplasia. The long-term results of complete repair and staged correction are comparable but are associated with a high probability of reoperation and re-intervention.

The importance of this case report is two fold. It provides an alternative treatment strategy to very high operative risk patients with IAA by palliating them using a hybrid approach. It also demonstrates safe insertion of the ductal sheath in the infundibulum, rather than the main pulmonary artery, providing ample distance to deploy the ductal stent without any adverse hemodynamic consequences of pulmonary valve insufficiency.

## CASE REPORT

A premature neonate male of 26 weeks gestational age weighing 1.35 kg was born with prenatal diagnosis of interrupted aortic arch and ventricular septal defect. He was started on prostaglandin therapy and mechanical ventilation shortly after an uneventful delivery. The postnatal echocardiogram confirmed the prenatal diagnosis of type B interrupted aortic arch with posteriorly malaligned conoventricular ventricular septal defect and left ventricular outflow tract obstruction. The aortic valve appeared bicuspid with moderate annular hypoplasia and mild hypoplasia of the ascending aorta. An aberrant right subclavian artery arose from proximal descending aorta, and a large patent ductus arteriosus was confirmed. Spiral computed tomography (CT) scanning (Siemens Definition, Iselin, NJ) with angiography and 3-D image reconstruction confirmed the diagnosis. The patient subsequently developed necrotizing enterocolitis with perforation of the distal ileum, requiring ileocolectomy with an end-ileostomy. He received intravenous total parenteral nutrition for nutritional support and was treated with a full course of intravenous antibiotics for vancomycin-resistant Enterococcus (VRE) sepsis, while being concomitantly treated with maximal medical therapy for severe pulmonary over-circulation. Given multiple comorbidities, he was considered a substantial risk for a complete cardiac repair and appeared to be a more suitable candidate for a hybrid procedure using ductal stenting with bilateral branch pulmonary artery banding.

**Figure 1:**
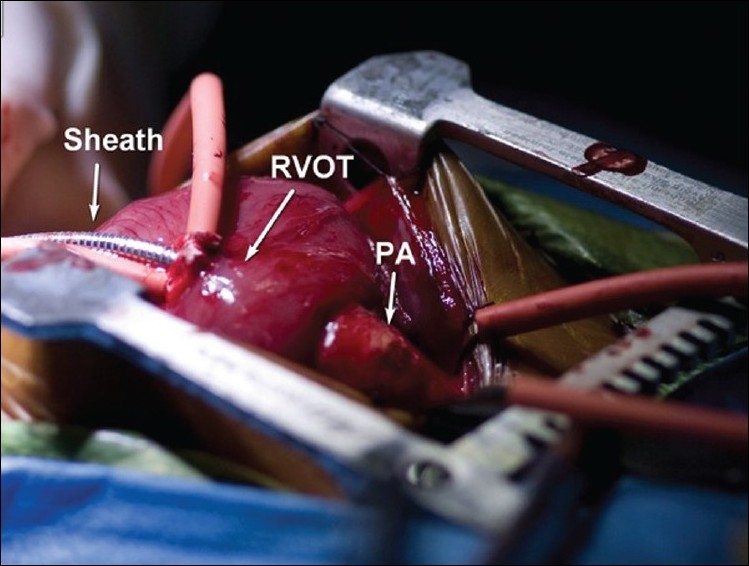
Intra-operative photograph of the introducer sheath for placement of ductal stent (RVOT= right ventricular outflow tract, PA= pulmonary artery)

**Figure 2:**
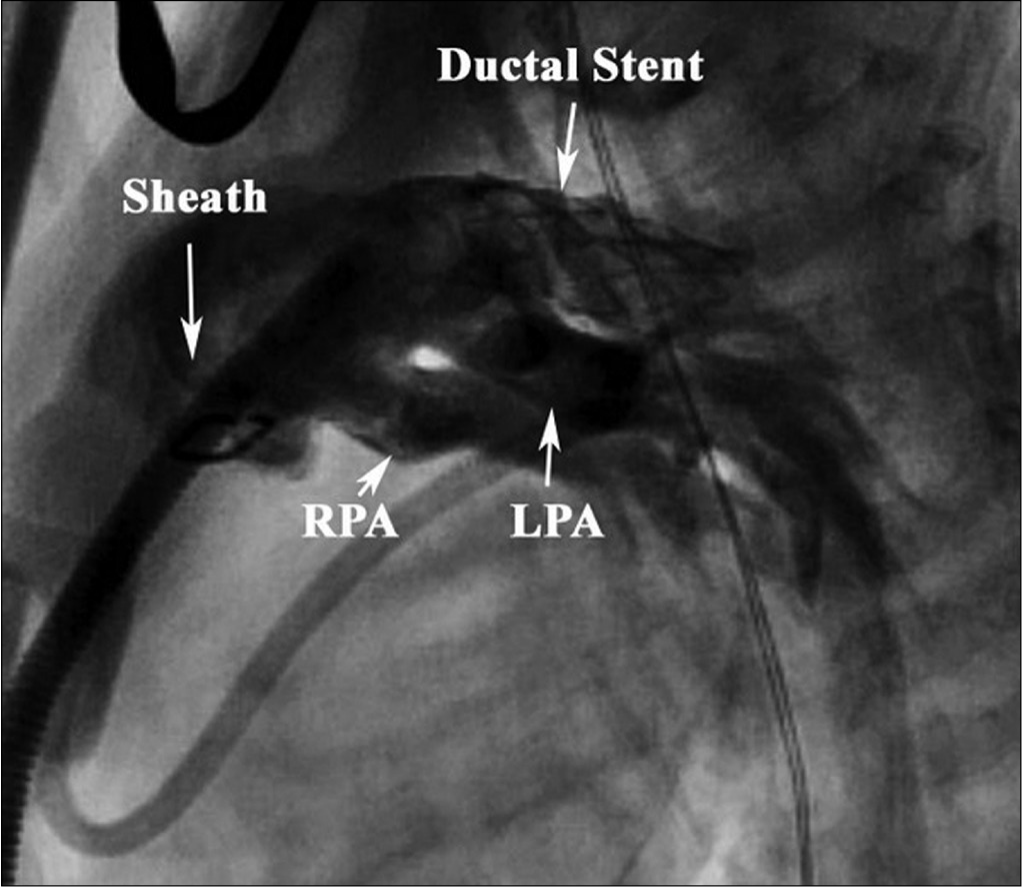
Angiographic image of ductal stent and branch pulmonary arteries (RPA= right pulmonary artery, LPA= left pulmonary artery)

The hybrid procedure was performed in a hybrid cardiac catheterization suite with cardiopulmonary bypass on standby. Using a midline sternotomy, the left and right branch pulmonary arteries were mobilized at their origin. After full intravenous heparinization, a pledgeted purse-string suture was placed in the infundibulum of the right ventricular outflow tract about 1 cm below the pulmonary annulus. A 7Fr/11-cm wire-reinforced introducer sheath (Arrow^®^ Super Arrow Flex^®^; Reading, PA) was introduced through the purse-string suture into the proximal main pulmonary artery [Figure 1]. A guide wire was inserted through the sheath and passed into the ductus arteriosus and the descending aorta under fluoroscopic guidance. A 7×18-mm Palmaz Genesis factory-mounted balloon expandable stent (Johnson and Johnson/Cordis, Genesis PG1870BSS; Miami Lakes, FL) was deployed in the ductus, preserving the origin of the left and right subclavian arteries [Figure 2].

The branch pulmonary arteries were banded by securing a 5 mm long segment of an opened 4-mm expanded PTFE graft around each branch pulmonary artery. The systemic saturation ranged between 80% and 90% while the patient was ventilated with 50% inspired oxygen. The immediate postoperative echocardiogram revealed normal bi-ventricular function, proper placement of the ductal stent and pulmonary artery bands with 30-35 mmHg pressure gradients across the bands bilaterally. The patient was successfully extubated on postoperative day 2, had appropriate and steady weight gain in the early postoperative period and was subsequently discharged on digoxin and furosemide. He has been followed up very closely for the last 6 months since his hybrid procedure and remains in balanced circulation with steady somatic growth.

## DISCUSSION

Interrupted aortic arch (IAA) is a complex congenital abnormality characterized by absence of anatomical continuity of the aortic arch with the aortic isthmus. It is associated with a wide variety of intracardiac malformations, most commonly a ventricular septal defect in addition to left ventricular outflow tract obstruction and arch hypoplasia. Despite marked improvement in the outcomes for many congenital heart defects, repair of interrupted aortic arch remains associated with a high operative mortality and poor long-term outcome. A multi-institutional study was conducted to look at the independent risk factors contributing to the poor outcome of some variants of this malformation.[1] The presence of low birth weight, left ventricular outflow tract obstruction, and aortic annular and arch hypoplasia were among the most important factors contributing to the high operative mortality and poor outcome.[1,2] The results of one-stage and two-stage repair have improved over the last several years and have been comparable.[3,4] However, the long-term probability of reoperation and re-intervention remains high regardless of operative technique, with the associated anomalies playing an important role in the outcome.[3,4]

The hybrid procedure has been utilized at various centers for the treatment of complex uni-ventricular defects such as hypoplastic left heart syndrome, particularly in high-risk situations.[5] We considered using the hybrid approach for this bi-ventricular heart as a means to ameliorate the associated high-risk factors without exposing the patient to the additional risks of cardiopulmonary bypass, cardioplegic arrest and hypothermic circulatory arrest.

The preoperative workup involved using computed tomography (CT) with 3-D reconstruction of the heart and great vessels. The CT was very helpful in planning and orchestrating the operative and interventional approach. The CT images provided information on the arch anatomy, the size of the arterial duct and relationship of the vascular structures to the arch and great vessels.

In the typical hybrid procedure, the arterial duct is usually accessed through a sheath placed in the main pulmonary artery just proximal to the branch pulmonary arteries.[5] However, this approach allows for only a few millimeters of the sheath to be inserted into the main pulmonary artery during placement of the stent into the arterial duct. To provide more flexibility and safety, we inserted the sheath through the pulmonary infundibulum, about 1 cm below the pulmonary annulus, and secured it with a purse-string suture. We believe this technique avoids inadvertent injury and distortion of the pulmonary valve by the sheath, and provides adequate distance from the tip of the sheath to the arterial duct in order to safely and accurately deploy and dilate the stent. The potential disadvantage of such maneuver is the possible mild-to-moderate pulmonary regurgitation, which might affect systemic perfusion during the procedure which was tolerated well in this case.

The hybrid approach was very useful in this case to palliate a high-risk patient in severe pulmonary over-circulation not responsive to optimal medical management. The patient achieved a well-balanced circulation with dramatic improvement of his heart failure. Finally, palliating this patient using the hybrid approach allowed for a more accurate assessment on the suitability of a bi-ventricular versus uni-ventricular repair.

The downside of the hybrid procedure is the future need to perform the arch reconstruction in the presence of ductal stent, and pulmonary arterioplasty on both branch pulmonary arteries at the time of de-banding. Nonetheless, the operative repair should be better tolerated in a well-developed infant with a protected pulmonary vascular bed and void of congestive heart failure.

A successful hybrid palliative approach was used in a high-risk neonate with interrupted aortic arch and ventricular septal defect. This is a safe and reproducible approach that can be considered in high-operative-risk neonates with multiple concomitant risk factors. It allows for immediate resolution of the congestive heart failure and provides time for recovery and growth for future operative repair. The right ventricular infundibulum was used as the insertion site for ductal sheath, which provided a reliable and safe conduit for stent placement with no adverse hemodynamic consequence during and after the procedure.

## References

[1] Jonas RA, Quaegebeur JM, Kirklin JW, Blackstone EH, Daicoff G (1994). Outcomes in patients with interrupted aortic arch and ventricular septal defect: A multiinstitutional study. J Thorac Cardiovasc Surg.

[2] Oosterhof T, Azakie A, Freedom RM, Williams WG, McCrindle BW (2004). Associated factors and trends in outcomes of interrupted aortic arch. Ann Thorac Surg.

[3] Brown JW, Ruzmetov M, Vijay P, Rodefeld MD, Turrentine MW (2006). Outcomes in patients with interrupted aortic arch and associated anomalies: A 20-year experience. Eur J Cardiothorac Surg.

[4] Schreiber C, Eicken A, Vogt M, Günther T, Wottke M, Thielmann M (2000). Repair of interrupted aortic arch: Results after more than 20 years. Ann Thorac Surg.

[5] Pizarro C, Murdison KA, Derby CD, Radtke W (2008). Stage II Reconstruction after hybrid palliation for high-risk patients with a single ventricle. Ann Thorac Surg.

